# Benchmarking the use of blood products in cardiac surgery to stimulate awareness of transfusion behaviour: Results from a four-year longitudinal study

**DOI:** 10.1007/s12471-016-0936-1

**Published:** 2016-12-16

**Authors:** C. Brouwers, B. Hooftman, S. Vonk, A. Vonk, W. Stooker, W. H. te Gussinklo, R. M. Wesselink, C. Wagner, M. C. de Bruijne

**Affiliations:** 1 EMGO+ Institute for Health and Care Research, Department of Public and Occupational Health, VU University Medical Center, Amsterdam, The Netherlands; 2 Department of Cardiothoracic Surgery, VU University Medical Center, Amsterdam, The Netherlands; 3 Department of Cardiothoracic Surgery, Onze Lieve Vrouwe Gasthuis, Amsterdam, The Netherlands; 4 Department of Cardiothoracic Surgery, Academic Medical Center, Amsterdam, The Netherlands; 5 Department of Anesthesiology and Intensive Care, St. Antonius Hospital, Nieuwegein, The Netherlands; 6 Netherlands institute for health services research (NIVEL), Utrecht, The Netherlands

**Keywords:** Cardiac surgery, CABG, Valve, Blood transfusion, Blood products, Benchmark

## Abstract

**Introduction:**

Cardiac operations account for a large proportion of the blood transfusions given each year, leading to high costs and an increased risk to patient safety. Therefore, it is important to explore initiatives to reduce transfusion rates. This study aims to provide a benchmark for transfusion practice by inter-hospital comparison of transfusion rates, blood product use and costs related to patients undergoing coronary artery bypass grafting (CABG), valve surgery or combined CABG and valve surgery.

**Methods:**

Between 2010 and 2013, patients from four Dutch hospitals undergoing CABG, valve surgery or combined CABG and valve surgery (*n* = 11,150) were included by means of a retrospective longitudinal study design.

**Results:**

In CABG surgery the transfusion rate ranged between 43 and 54%, in valve surgery between 54 and 67%, and in combined CABG and valve surgery between 80 and 88%. With the exception of one hospital, the trend in transfusion rate showed a significant decrease over time for all procedures. Hospitals differed significantly in the units of blood products given to each patient, and in the use of specific transfused combinations of blood products, such as red blood cells (RBCs) and a combination of RBCs, fresh frozen plasma (FFP) and platelets.

**Conclusion:**

This study indicates that benchmarking blood product usage stimulates awareness of transfusion behaviour, which may lead to better patient safety and lower costs. Further studies are warranted to improve awareness of transfusion behaviour and increase the standardisation of transfusion practice in cardiac surgery.

## Introduction

The use of transfused blood products is disproportionately distributed among hospitalised patients, whereby a minority of patients consume the majority of transfused blood products [1]. Patients undergoing cardiac procedures are more likely to receive transfused blood products because of the high risk of blood loss and severe anaemia [2]. Clinical guidelines regarding the use of blood products in cardiac surgery have been available since the mid-1980s and support surgeons in their choice for blood transfusion by providing recommendations regarding preoperative risk management, perioperative blood conservation, and the management of blood resources [1, 3, 4].

However, despite these guidelines there is a great variability in the use of blood products between countries, institutions and practitioners in cardiac surgery, which are caused by differences in human, technical and organisational-related factors [5, 6]. One way of examining the differences in these factors between hospitals is by benchmarking. Benchmarking is the process of establishing a standard of excellence by comparing a particular activity and its outcomes in one organisation with the same activity in another organisation [7].

This study aims to provide a benchmark for transfusion practice by comparing transfusion rates, blood product use and costs in patients undergoing coronary artery bypass grafting (CABG), valve surgery or combined CABG and valve surgery between hospitals.

## Materials and methods

### Study setting and population

This retrospective longitudinal study was carried out in two academic hospitals and two non-academic top clinical centres in the Netherlands. Hospital data on all patients undergoing CABG, valve surgery or combined CABG and valve surgery between 2010 and 2013 was obtained from each hospital. Patients under 18 years of age, patients admitted before or after the study period, patients whose period of hospitalisation overlapped two calendar years, and patients undergoing CABG or valve surgery in combination with complex cardiothoracic surgery were excluded from the study.

The data for this study were not collected simultaneously, instead we collected the data in three steps: in 2011 we collected the data from 2010, in 2012 we collected the data from 2011, and in 2014 we collected the data from 2012 and 2013. After each round of data collection, the hospitals received a report in which the transfusion rates and costs between the hospitals were compared. In addition, in May 2012 and September 2013 benchmark meetings were organised which were attended by representatives from each hospital who were involved in blood transfusion practice in cardiac surgery patients. At these meetings, the results of the report were discussed in order to share experiences, create awareness, and to stipulate reduction strategies for the coming year.

This study was approved by the medical ethics committee of the VU University Medical Center, Amsterdam.

### Data collection

Data on sex, age, type of surgery (CABG or valve surgery), blood product use for each patient throughout the hospital admission period (none, red blood cells (RBCs), fresh frozen plasma (FFP) or platelets), length of hospital stay (in days), and haemoglobin (i. e. preoperative Hb, lowest intraoperative Hb and postoperative Hb) were collected using the electronic health record system of the hospitals. Data on blood loss was collected using the clinical ward system of cardiothoracic surgery. Blood loss was defined as chest tube blood loss at 24 h postoperatively.

### Costs

The costs of blood products were based on the average cost price per unit from Sanquin Blood Supply between 2010 and 2013. These were € 216.50 for RBCs, € 185.70 for FFP and € 521.90 for platelets. The platelet product used for the cost calculation is composed of five buffy coats of identical ABO and Rh(D) compatible blood groups, mixed with plasma or platelet storage solution.

### Data analysis

To compare the demographic and clinical characteristics of the patients in each hospital, one-way ANOVA, Kruskal-Wallis tests, or Chi-squared tests were used depending on the measurement and variable distribution. Chi-squared tests were also used to examine the differences in transfusion rates over time and between hospitals. Transfusion rates were defined as the proportion of patients receiving any transfusion with packed RBCs, FFP and/or platelets. Combinations of transfused blood products were defined as the percentage of patients receiving at least one unit of RBCs, FFP or platelets, or a combination of these types of blood product.

A logistic regression analysis was performed to investigate the predictors of blood transfusion. The following covariates were selected for inclusion in the regression analysis: hospital (A–D), age during hospitalisation, blood loss at 24 h, type of surgery (CABG, valve, CABG + valve), total duration of hospitalisation, and haemoglobin levels before surgery. All these variables were entered into the logistic regression model simultaneously using the enter method. Missing value analysis showed that hospital D had no data on blood loss at 24 h in 2011. Hence, the patients from hospital D from 2011 were excluded from the analyses. For the other variables only a small percentage of values (≈5%) was missing at random.

The mean costs of transfused blood products per cardiothoracic surgical procedure were calculated by multiplying cost prices by the mean number of units transfused (based on both transfused and non-transfused patients). The difference in costs between hospitals were calculated using a one-way ANOVA. A sensitivity analysis showed no difference in results between the one-way ANOVA with and without bootstrapping (using 1000 bootstrap samples). The maximum cost difference for each cardiothoracic surgical procedure between the hospitals was calculated by subtracting the lowest mean costs from the highest mean costs. A *p*-value of <0.05 was considered to be statistically significant. The data were analysed with SPSS version 20.0 software (IBM, New York, USA).

## Results

### Patient characteristics

The total number of patients undergoing CABG, valve surgery or combined CABG and valve surgery between 2010 and 2013 was *n* = 11,150, of which 57% (*n* = 6359) were CABG procedures, 28% (*n* = 3146) valve procedures and 15% (*n* = 1645) combined CABG and valve procedures. The majority of valve procedures were aortic valve replacements (*n* = 2778, 52%), followed by mitral valve repairs (*n* = 1013, 19%), multiple valve repairs (*n* = 969, 17%) and other (*n* = 624, 12%).

There were significant differences between the hospitals in the following: patients’ gender (*p* < 0.001), age (*p* = 0.019), preoperative Hb values (*p* < 0.001), lowest intraoperative Hb values (*p* < 0.001), discharge Hb values (*p* < 0.001), 24-hour blood loss (*p* < 0.001), and total and postoperative stay in hospital (*p* < 0.001 and *p* = 0.001, respectively) (Table 1).

**Table 1:** Patient demographics and surgical characteristics (2010–2013)

	Hospital A	Hospital B	Hospital C	Hospital D^a^	*p*-value
*N*	2356	2610	2382	3802	
*Males (%)*	75%	71%	71%	70%	<0.001
*Age (years)*	67.3 ± 10.1	67.4 ± 9.9	66.6 ± 11.3	67.3 ± 10.6	0.019
*Type of surgery*					<0.001
CABG [*n*]	1516	1427	1291	2125	
Valve surgery [*n*]	512	723	736	1175	
CABG + valve surgery [*n*]	328	460	355	502	
*Type of valve surgery*					<0.001
Aortic valve replacement [*n*]	454	681	681	962	
Mitral valve repair [*n*]	131	225	107	550	
Other [*n*]	464	49	101	10	
Multiple valves [*n*]	395	217	202	155	
*Preoperative Hb (mmol/l)*	8.6 ± 1.0	8.0 ± 1.1	8.3 ± 1.2	8.3 ± 1.1	<0.001
*Lowest intraoperative Hb (mmol/l)*	6.5 ± 0.9	5.2 ± 0.8	5.1 ± 1.1	–	<0.001
*Discharge Hb (mmol/l)*	7.0 ± 0.9	6.6 ± 0.9	6.4 ± 0.8	6.7 ± 0.9	<0.001
*24-hour blood loss (ml)*					
CABG	486 (295–560)	415 (310–610)	800 (575–1150)	710 (540–940)	<0.001
Valve surgery	315 (225–490)	355 (250–575)	550 (360–830)	600 (400–920)	<0.001
CABG + valve surgery	495 (350–781)	535 (360–802)	900 (610–1305)	1175 (800–1750)	<0.001
*Hospital length of stay (days)*	10.3 ± 9.1	11.6 ± 9.1	10.8 ± 9.0	10.7 ± 8.8	<0.001
*Postoperative length of stay (days)*	6.8 ± 8.7	7.8 ± 6.4	7.1 ± 5.5	7.7 ± 7.0	0.001

^a^Hospital D has missing data on 2011

*CABG* coronary artery bypass graft, *Hb* haemoglobin

### Transfusion rates

**Fig. 1:**
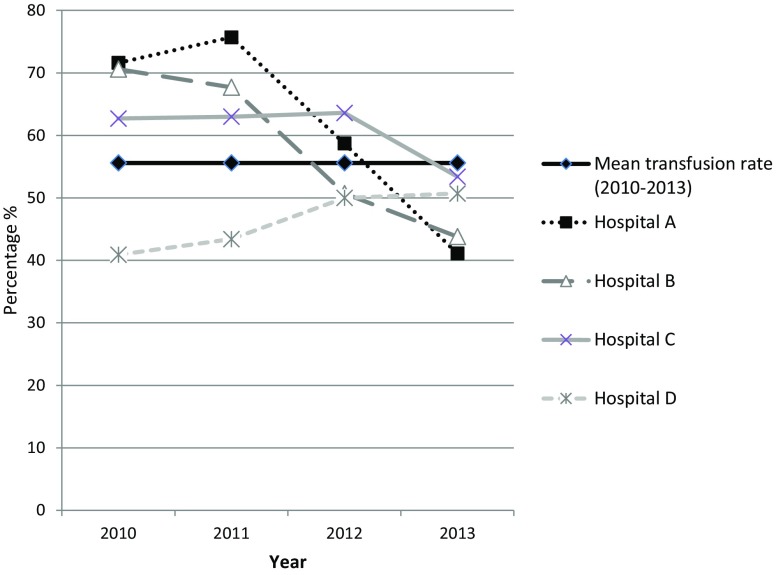
Trend in transfusion rate of between 2010 and 2013 (CABG, valve surgeries and combined CABG + valve surgeries) (*CABG* coronary artery bypass graft)

Between 2010 and 2013 the overall transfusion rate of patients undergoing CABG surgery, valve surgery or combined CABG and valve surgery was 56.9%. The trend of the transfusion rate between 2010 and 2013 for CABG surgery, valve surgery and CABG + valve surgery is shown in Fig. 1. When stratified by type of surgery, the transfusion rate in CABG surgery ranged between 43 and 54%, in valve surgery between 54 and 67%, and in combined CABG and valve surgery between 80 and 88%.

In hospitals A and B the transfusion percentage decreased significantly for all surgical procedures over time (CABG: *p* < 0.001, valve: *p*’s < 0.001, CABG + valve: *p*’s ≤ 0.02, in both hospitals). In hospital C the transfusion rate for CABG surgery (*p* = 0.03) and combined CABG and valve surgery (*p* = 0.006) decreased significantly, while the transfusion rate of valve surgery did not (*p* = 0.26). In hospital D there was no significant decrease in the transfusion rate of CABG (*p* = 0.79), valve (*p* = 0.74), and combined CABG and valve surgery (*p* = 0.12).

### Predictors of blood transfusion

Multivariate logistic regression analyses were performed to examine the predictors of blood transfusion. The site (hospital B vs. hospital A, OR = 0.43; CI = 0.37–0.50, *p* < 0.001; hospital C vs. hospital A, OR = 0.27; CI = 0.22–0.33, *p* < 0.001; hospital D vs. hospital A, OR = 0.16; CI = 0.14–0.19, *p* < 0.001), female gender (OR = 2.73; CI = 2.40–3.10, *p* < 0.001), age (OR = 1.08; CI = 1.01–1.10, *p* < 0.001), duration of hospitalisation (OR = 1.09; CI = 1.08–1.12, *p* < 0.001), preoperative Hb value (OR = 0.57; CI = 0.54–0.60, *p* < 0.001), blood loss at 24 h (OR = 1.01; CI = 1.00–1.02, *p* < 0.001), and type of surgery (valve (OR = 1.86; CI = 1.64–2.11, *p* < 0.001); and combined CABG and valve surgery, OR = 2.96; CI = 2.47–3.55, *p* < 0.001) were all significantly associated with the risk of receiving a transfusion (*p*’s < 0.001). These variables explained 44% of the model variance.

### Blood product use

There were significant differences between the hospitals in the median number of transfused units of RBC, FFP and platelets per patient in CABG, valve surgery or combined CABG and valve surgery (*p* < 0.001, *p* = 0.001 and *p* = 0.023, respectively). Combining all types of surgery, the majority of patients (47%) received at least one transfusion with RBCs, 16% received more than two units, and only 8% received more than four units. For FFP and platelets, 33% and 10% of the patients received at least one unit, and 8% and 1% more than two units, respectively.

### Combinations of transfused blood products

**Fig. 2:**
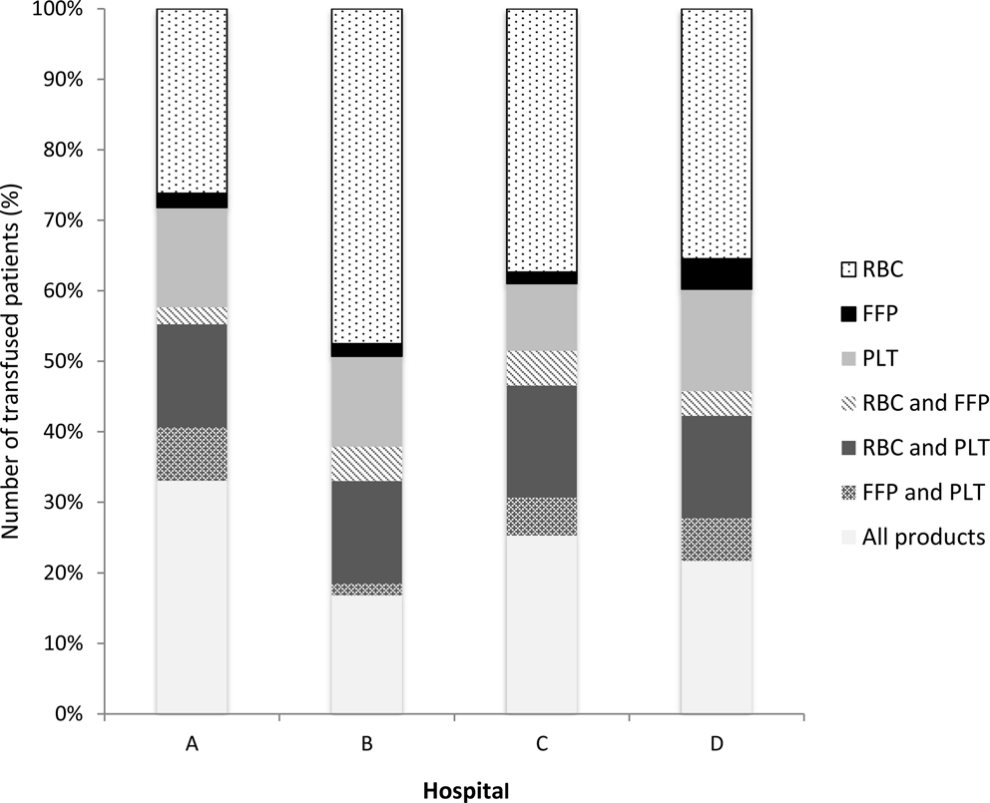
Relative number of patients with transfused RBCs, FFP, platelets or combination of these type of blood products in CABG surgery (*CABG* coronary artery bypass graft, *RBC* red blood cells, *FFP* fresh frozen plasma, *PLT* platelets)

**Fig. 3:**
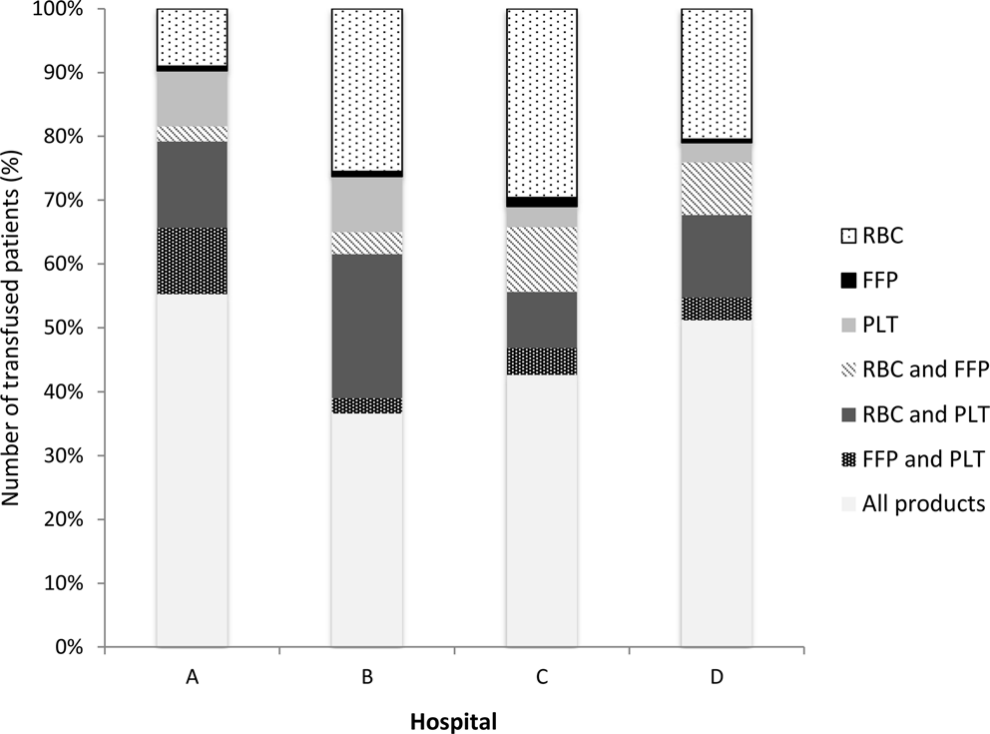
Relative number of patients with transfused RBCs, FFP, platelets or combination of these type of blood products in valve surgery (*RBC* red blood cells, *FFP* fresh frozen plasma, *PLT* platelets)

**Fig. 4:**
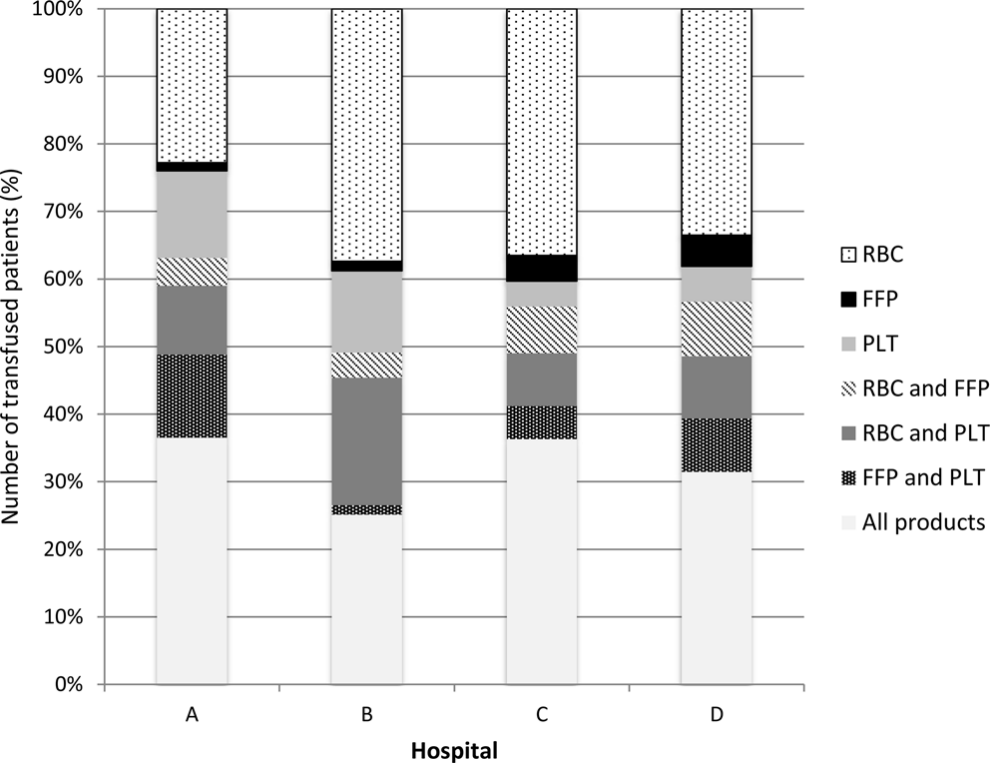
Relative number of patients with transfused RBCs, FFP, PLTs or combination of these type of blood products in CABG combined with valve surgery (*CABG* coronary artery bypass graft, *RBC* red blood cells, *FFP* fresh frozen plasma, *PLT* platelets)

In CABG surgery, the main differences between the hospitals were in patients receiving RBCs only, ranging from 26.2% (hospital A) to 47.5% (hospital B), and a combination of RBCs, FFP and platelets, ranging from 16.8% (hospital B) to 33.1% (hospital A) (panel A) (Fig. 2). In valve surgery there is a large discrepancy between the use of RBCs between hospital A (9%) and hospital C (29.6%) (panel B) (Fig. 3). Combined CABG and valve surgery shows a similar pattern when compared with CABG surgery and is also associated with more hospital-specific combinations of RBCs, FFP and platelets (panel C) (Fig. 4). The proportion of transfused blood product combinations remained similar between 2010 and 2013 for each hospital (not shown).

### Costs of blood transfusion

Between 2010 and 2013 the mean costs per patient between the hospitals ranged from € 174 to € 827 for RBCs, € 49 to € 374 for FFPs, € 156 to € 550 for platelets (Table 2). The mean costs for the total use of RBCs, FFP and platelets combined per patient ranged between € 407 (hospital D) to € 1673 (hospital C). The maximum cost difference per cardiothoracic surgical procedure was € 291 (€ 698 − € 407) for CABG, € 459 (€ 1131 − € 672) for valve surgery, and € 312 (€ 1673 − € 1361) for CABG combined with valve, which corresponds to a maximum cost difference of 42% per CABG surgery and valve surgery, and 19% for combined CABG and valve surgery. When examining the change in costs per hospital over time, hospitals A and B show a significant reduction in the total costs (RBC, FFP and platelet) for CABG, valve and combined CABG and valve surgery (*p*’s < 0.002) between 2010 and 2013. Hospitals C and D did not show a decrease in total costs for CABG, valve and combined CABG and valve surgery over time (*p* = 0.14 and *p* = 0.60, respectively).

**Table 2:** Costs (in Euros) of blood products per operation category (2010–2013)^a^

Variables	Hospital A	Hospital B	Hospital C	Hospital D	*p*-value
Total costs 2010–2013	€ 1.949.550	€ 1.938.883	€ 2.328.219	€ 2.380.796	
**Mean costs per surgery** ^b^					
*RBCs*					
CABG	282 (252–311)	252 (221–284)	335 (305–366)	*174 (158–190)*	<0.001
Valve	362 (299–424)	495 (410–579)	519 (449–590)	*313 (274–353)*	<0.001
CABG & valve	700 (586–814)	751 (633–840)	827 (712–942)	*679 (611–784)*	0.22
*FFP*					
CABG	120 (107–133)	*49 (41–58)*	129 (112–147)	68 (60–76)	<0.001
Valve	201 (172–229)	*118 (87–148)*	297 (238–355)	148 (129–167)	<0.001
CABG & valve	342 (296–388)	*177 (134–220)*	374 (314–434)	286 (255–317)	<0.001
*PLTs*					
CABG	242 (221–262)	*156 (136–176)*	234 (210–257)	165 (150–179)	<0.001
Valve	319 (293–354)	300 (259–340)	315 (265–366)	*210 (180–241)*	<0.001
CABG & valve	550 (489–612)	*432 (382–482)*	472 (394–550)	483 (436–530)	<0.001
*RBCs, FFP and PLTs*					
CABG	644 (589–698)	458 (405–511)	698 (638–759)	*407 (376–438)*	<0.001
Valve	881 (769–993)	912 (768–1056)	1131 (969–1293)	*672 (590–754)*	<0.001
CABG & valve	1593 (1397–1789)	*1361 (1171–1551)*	1673 (1438–1908)	1448 (1324–1572)	0.08

^a^Data are reported as mean (95% CI) euros calculated on total study population (both transfused and non-transfused patients), costs indicated in bold are the lowest costs for that group for all hospitals

^b^The costs of blood products are based on the average cost prizes per unit of Sanquin Blood Supply between 2010–2013 (Amsterdam, the Netherlands), which were € 213.50 for RBCs, € 183.35 for FFP and € 511.25 for PLTs

*CABG* coronary artery bypass graft, *RBC* red blood cells, *FFP* fresh frozen plasma, *PLT* platelets

## Discussion

The results of this study indicate that there are substantial differences between the hospitals in the relative number of patients receiving a blood transfusion. With the exception of one hospital, the trend in transfusion rate showed a decrease over time for all types of surgery. Although we cannot assume a direct causal relationship between the benchmark study and this decrease in transfusion rate, it seems plausible that the benchmark helped in creating more awareness on transfusion practices, and motivated hospitals to initiate strategies to improve transfusion practices. Optimisation of blood transfusion was achieved by the use of cell saver, continuous availability of thromboelastometry (ROTEM, TEM Systems Inc.), protamine management, prevention of haemodilution, fibrinogen concentrates and prothrombin complex concentrates, intravenous administration of iron preoperatively in patients with iron deficiency anaemia, and careful haemostasis. These strategies were implemented in all four hospitals. In addition, hospital A stated it used perioperative normothermia and has a strong focus on optimising surgical technique to prevent blood loss. Hospital B used retrograde autologous priming of the bypass circuit and focused on optimising the transfer from the operating room to the intensive care unit.

This study identified differences between hospitals in sociodemographic and clinical parameters, such as age, gender, perioperative and postoperative Hb and blood loss. The exact reasons for these differences are unknown. Also in the literature the difference in blood loss between hospitals remains largely unexplained, irrespective of differences in the measurement of blood loss or patient characteristics [8]. Vonk et al. stated that a decrease in postoperative blood loss in a hospital was probably due to the introduction of cell salvage [9]. Although a difference in the timing of implementation of cell salvage between individual hospitals could explain the difference in blood loss, it seems more likely that the differences are the result of perioperative blood management (protocols, logistical differences, higher tolerance of blood loss to prevent clotting complications, use of alternatives for FFP). In relation to Hb, we know that hospitals C and D accepted a lower perioperative Hb and a lower postoperative discharge Hb than hospitals A and B, resulting in a higher transfusion threshold. The latter strengthens the idea that there is a need for more consensus on the appropriate Hb transfusion threshold.

This study also showed hospital-specific transfusion practices such as the use of hospital-specific transfused combinations and the number of RBC, FFP and platelet units used per patient. The percentage of patients receiving at least one, two and four units of RBCs was slightly lower in comparison with previous findings [10, 11]. Hospital-specific transfusion practices were also identified in previous studies [12, 13]. An explanation for this finding could be a difference in transfusion behaviour, in which the likelihood of receiving a blood transfusion is more associated with the physician’s tolerance level of anaemia than with the patient’s actual physiological need for correction of the anaemia [14]. This could lead to inconsistent transfusion practices and even inappropriate transfusions [15]. Alternatively, the hospital-specific transfusion practices we identified could also be related to the difference in patient mix between academic and top clinical hospitals, or due to the fact that there is no clear consensus on transfusion triggers between hospitals [16–18].

In this study we also examined the costs associated with the use of blood products in CABG, valve surgery and combined CABG and valve surgery. This study indicates that improving transfusion practices may result in considerable cost reduction. This is important as these financial resources can then be used to improve other aspects of patient care.

### Limitations

The results of the current study should be interpreted with the following limitations in mind. Despite our efforts to select homogenous samples of cardiac surgery patients there were still significant differences in demographic and surgical characteristics of the patients between the hospitals. These differences could have affected transfusion rates. Then again, we found a similar trend between the transfusion rate and increasing age, and between the transfusion rate and men vs. women (higher transfusion rate in women) between hospitals, meaning that these differences are unlikely to be responsible for the differences in transfusion behaviour. In addition, we were not able to correct for other differences in patient mix such as disease severity (i. e. EuroScore) and comorbidity. Also, this study did not collect information on possible wastage of blood products after the products had been issued from the blood transfusion laboratory, and on the use of alternatives for FFP, such as fibrinogen concentrate, recombinant factor VIIa (NovoSeven, Novo Nordisk Inc.) and 4‑factor prothrombin complex. Finally, our study did not link the use of transfused blood products to the health outcomes of the patients, such as number of re-operations and survival after surgery. However, other studies have shown that blood transfusion is associated with increased risk of morbidity and mortality [2, 18, 19].

### Recommendations

To successfully change transfusion practices it is crucial that there is initiative and commitment, not only by the department of cardiothoracic surgery but also by other disciplines involved in administering or handling blood products for transfusion. Such improvement strategies could include audits, multidisciplinary consensus meetings on blood transfusion practices, or the provision of insight into the use of blood products by the individual clinician [20]. Another improvement strategy to reduce the number of units of RBCs is the introduction of a new logistical policy of blood transfusion in which no elective RBC units are preoperatively ordered [21, 22].

In addition, by benchmarking with other hospitals, hospitals can share their experience and exchange ideas for improving the standardisation and frequency of blood transfusions. In order to further fine tune the transfusion practices in hospitals, it is also important to directly link blood transfusion practices to clinical outcomes in order to detect if changes in transfusion practices actually improve survival. Finally, further research is warranted to unravel the explanatory factors underlying the variation in use of blood products by gaining more insight into hospital-specific transfusion triggers, the transfusion triggers for each blood product, and by taking into account more variables related to patient characteristics and clinical data. Also, to attain a complete overview of current blood transfusion practices, it is important to expand benchmarking studies across more hospitals around the country, and to carefully monitor improvements or deteriorations in each hospital.

## Conclusion

Benchmarking transfusion practices seems to be an effective way to improve awareness and increase the standardisation of transfusion practices in cardiac surgery. In addition, this study indicates that there are significant discrepancies in transfusion rates, the use of specific blood products, and in costs associated with CABG, valve, and combined CABG and valve surgery, which seem to decrease over time.
